# Excavations at Mlambalasi Rockshelter: a Terminal Pleistocene to Recent Iron Age Record in Southern Tanzania

**DOI:** 10.1007/s10437-017-9253-3

**Published:** 2017-05-27

**Authors:** K. M. Biittner, E. A. Sawchuk, J. M. Miller, J. J. Werner, P. M. Bushozi, P. R. Willoughby

**Affiliations:** 1 MacEwan University, Edmonton, Canada; 2 University of Toronto, Toronto, Canada; 3 University of Alberta, Edmonton, Canada; 4 University of Dar es Salaam, Dar es Salaam, Tanzania

**Keywords:** Tanzania, East Africa, Later Stone Age, Iron Age, Burials, Terminal Pleistocene

## Abstract

The Mlambalasi rockshelter in the Iringa Region of southern Tanzania has rich artifactual deposits spanning the Later Stone Age (LSA), Iron Age, and historic periods. Middle Stone Age (MSA) artifacts are also present on the slope in front of the rockshelter. Extensive, systematic excavations in 2006 and 2010 by members of the Iringa Region Archaeological Project (IRAP) illustrate a complex picture of repeated occupations and reuse of the rockshelter during an important time in human history. Direct dates on *Achatina* shell and ostrich eggshell (OES) beads suggest that the earliest occupation levels excavated at Mlambalasi, which are associated with human burials, are terminal Pleistocene in age. This is exceptional given the rarity of archaeological sites, particularly those with human remains and other preserved organic material, from subtropical Africa between 200,000 and 10,000 years before present. This paper reports on the excavations to date and analysis of artifactual finds from the site. The emerging picture is one of varied, ephemeral use over millennia as diverse human groups were repeatedly attracted to this fixed feature on the landscape.

## Résumé

L'abri sous roche Mlambalasi dans la région d'Iringa au sud de la Tanzanie est riche en artéfacts du Paléolithique supérieur jusqu'à l'âge du fer et la période historique. Des artéfacts du Paléolithique moyen sont présents sur le versant en face de l'abri. Des fouilles extensives et systématiques réalisées par l’équipe d’Iringa Region Archaeological Project (IRAP) révèlent une image complexe de l’utilisation et de la réutilisation de l'abri sous roche pendant un moment important dans l'histoire de l'humanité. Des datations directes de coquilles d'*Achatina* et de perles en coquilles d'autruches (OES) suggèrent que l’occupation la plus ancienne excavée à Mlambalasi, associée aux sépultures humaines, parvient d'une phase terminale du Pléistocène. Ceci est exceptionnel car les sites archéologiques, en particulier contenant des corps humains et d'autres matières organiques préservées, sont rares en Afrique subtropicale entre 200,000-10,000 années avant le présent. Cet article présente les fouilles effectuées jusqu'à ce jour ainsi que l'analyse des artéfacts trouvés sur le site. L'image qui ressort est celle d’une utilisation variée et éphémère à travers les millénaires au fur et à mesure que divers groupes ont été attirés à cet endroit permanent dans le paysage.

## Introduction

**Fig. 1:**
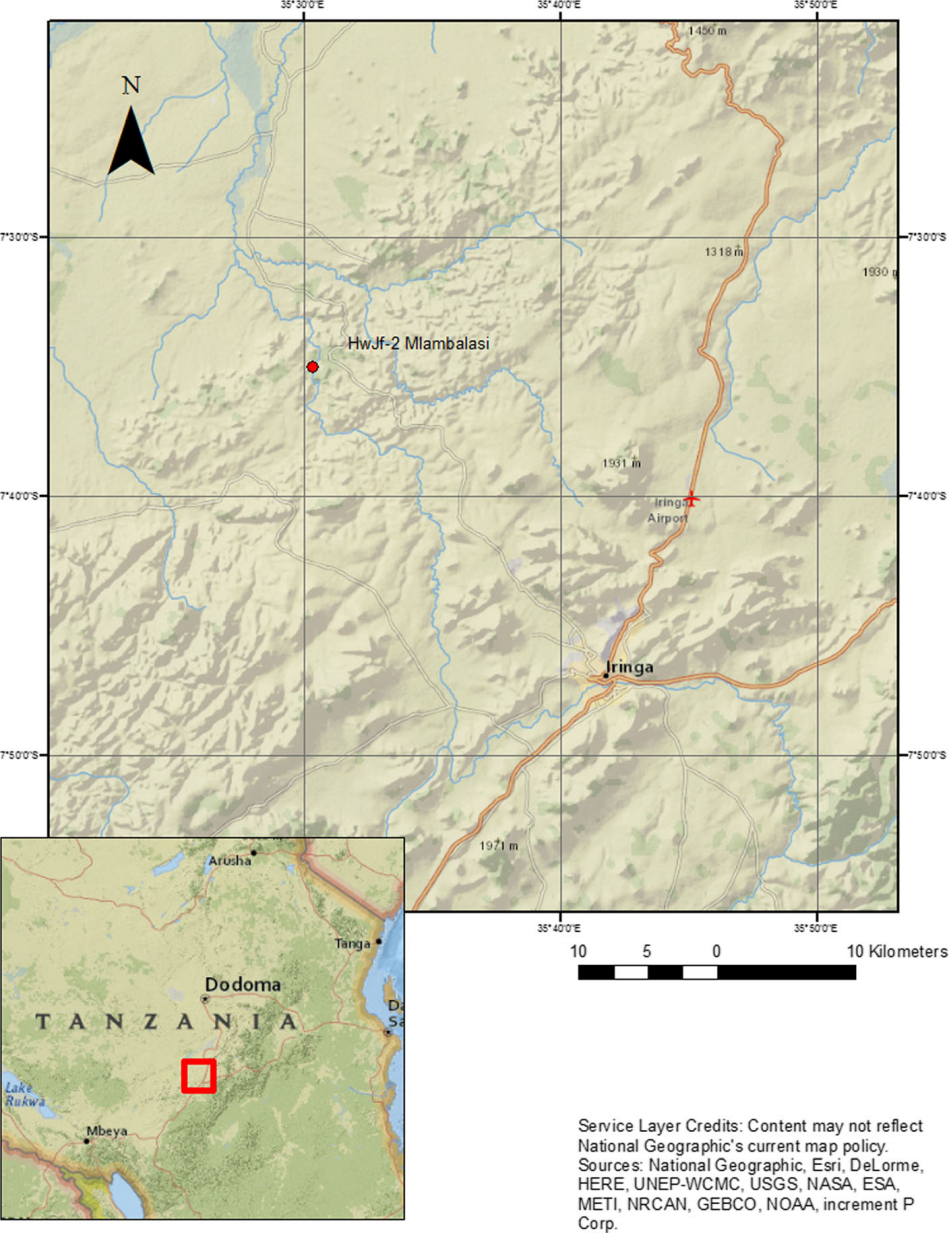
Location of Mlambalasi rockshelter

Although Tanzania has been an important center for archaeology and paleoanthropology since the 1940s, research has focused largely in the northern part of the country and along the Swahili coastline. This paper introduces inland research by the Iringa Region Archaeological Project (IRAP) at the Mlambalasi rockshelter site (HwJf-02). Located in the south-central highlands approximately 50 km away from the regional capital, Iringa City (Fig. 1), the site possesses a long, stratified sequence from the Pleistocene Later Stone Age (LSA) to the recent Iron Age and historic period.

Mlambalasi is better known for its historical significance. The rockshelter was the location of the last stand of Chief Mkwawa, the nineteenth-century paramount leader of the Hehe (Willoughby 2012). Mkwawa is said to have hid out in the rockshelter in 1898 to escape the German colonial army, eventually killing himself and his remaining servant to avoid capture. Mlambalasi rockshelter is just one part of the larger site complex, which also bears Mkwawa’s funeral monument and a historical information center. Mkwawa’s funeral monument, comprised of his tomb, the tomb of his servant, and an Uhuru (Freedom) memorial monument, is located a few meters below the main rockshelter.

**Fig. 2:**
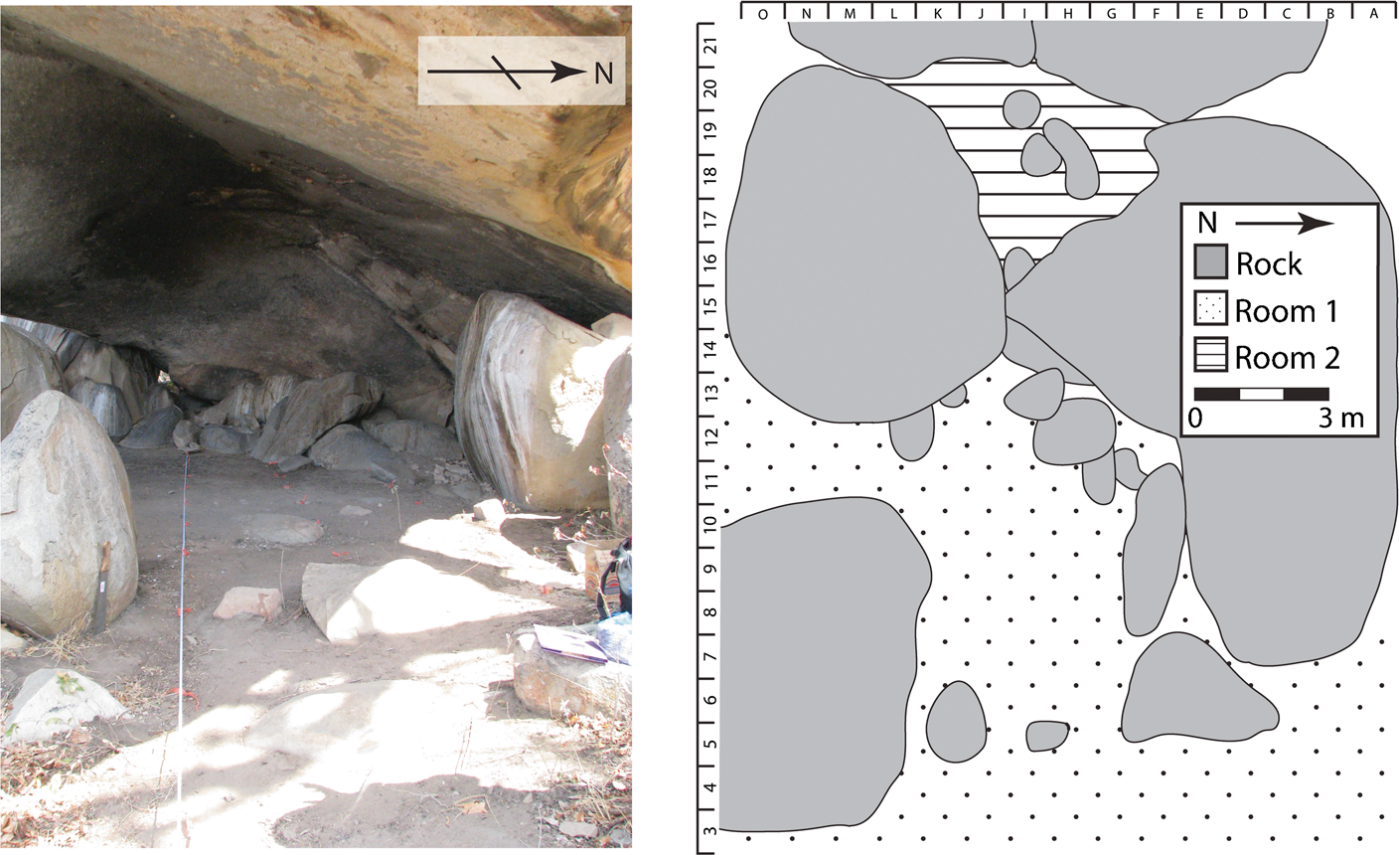
Mlambalasi rockshelter, rooms 1 and 2. *Image on the left* is a west-facing photograph of room 1; *right image* is a map showing rooms 1 and 2 (photo: J. Miller)

The main rockshelter is located a couple of meters up the incline of a large granitic outcrop, and is divided into two interconnected rooms (Fig. 2). Room 1 is approximately 12 × 8 m with a high roof creating an open, comfortable space. There are separate east and southwest entrances, with a large, granite boulder between them that probably originated from a roof collapse. In its current position, the boulder forms a partial wall that shields the interior from wind and conceals it on the landscape. Room 2 is approximately 4 × 4 m and can be accessed through a small crawl space or from its own west-facing entrance. Both rooms have abundant surface scatter including lithics, bone, iron slag, and pottery, as well as several large grindstones. The interior of the rockshelter and several nearby outcrops feature undated anthropomorphic rock art in red pigment, likely associated with Iron Age occupations.

Our team commenced archaeological excavations at the Mlambalasi rockshelter in July 2006 (Biittner et al. 2007). Initial test excavations revealed separate Iron Age and LSA occupations, including fragmentary human remains. Following this preliminary exploration, we learned that a Tanzanian research team had previously test-excavated the site in 2002 prior to the assignment of a SASES number. With this knowledge, IRAP returned to Mlambalasi in 2010 to locate and compare the 2002 and 2006 test units and continue investigation of human remains at the site. Our findings suggest repeated use and occupation of the rockshelter from approximately 20,000 years ago through modern times, with human burials dating back to the terminal Pleistocene.

## Background

The archaeological record at Mlambalasi offers rare insight into human adaptation and survival during an important but poorly understood time period. Very few sites span the transition from the Last Glacial Maximum (LGM) (c. 23,000–18,000 BP) to the beginning of the Holocene (c. 12,000 BP). Sites with preserved organic material, particularly human remains, are even scarcer (Grine 2016; Rightmire 1975; Stojanowski 2014). However, as recently noted by lithic specialists, there is very little archaeology of any type from this era (Pargeter and Brandt 2015).

This dearth of sites is likely related to a wide-scale reduction in human and animal habitats. Although free of ice sheets, glacial periods in Africa were marked by extremely arid conditions (Hamilton 1982). During the final stages of the Pleistocene, many African great lakes experienced significant water-level drops with related impacts on vegetation cover (Gasse 2000; Barker and Gasse 2003; Gasse et al. 2008; Vincens et al. 2005). Lake Victoria evaporated completely between c. 17,000–16,000 BP and again c. 15,000–14,000 BP, with a demonstrable “push-pull” effect on human and animal populations (Johnson et al. 1996; Tryon et al. 2015; Verheyen et al. 2003). Although we lack fine-grained palaeoclimatic data for many subregions (Grine 2016), the absence of archaeological sites in many parts of the continent during glacial maxima suggests that many regions could not sustain human life (Willoughby 2012).

Genetic studies, however, do not support an associated reduction in the human population during the LGM. This period appears to have been one of steady recovery after a proposed bottleneck event earlier in the Late Pleistocene (Gagneux et al. 1999; Harpending et al. 1993; Lahr and Foley 1998). Mitochondrial DNA studies of extant human groups indicate that our population began rapidly increasing during the Late Pleistocene LSA, instead of exclusively with the Neolithic spread of food production in the early-middle Holocene (Cox et al. 2009; Henn et al. 2012). This is consistent with evidence for growing technological innovation, regional exchange networks, and social complexity in the African LSA (McBrearty and Brooks 2000; Wadley 2014). The archaeological invisibility of these growing populations suggests that the problem may lie in where we are looking. If we wish to explore human adaptations at the end of the last glacial, we must turn our focus toward landscapes where humans and their records could survive.

Table 1 lists known archaeological sites dating to the terminal Pleistocene (c. 20,000–10,000 BP). We focus on sub-Saharan Africa, excluding large Epi-Palaeolithic cemeteries in the Maghreb and Nile Valley (e.g., Anderson 1968; Ferembach 1962) and southern African sites associated with the ancestors of the Khoesan (Grine et al. 2007, 2016; Houghton and Thackeray 2011; Pike et al. 2004; Walker 1995). The remaining sites from this time period are overwhelmingly located in rockshelter and cave deposits.

**Table 1:** Sub-Saharan archaeological sites that overlap with the Mlambalasi occupation c. 20,000–12,000 BP

Site	Country	Cave/rockshelter	Approximate date BP	Source
Matupi	DRC	X	>40,000–720 ± 45	Van Noten (1977), Van Noten and Cahen (1982)
Mumba	Tanzania	X	36,900 ± 800–12,000 ± 1700	Gliganic et al. (2012), Mehlman (1989)
Shum Laka	Cameroon	X	31,700 ± 750–9880 ± 100	Cornelissen (2003)
Kisese II*	Tanzania	X	31,480 + 1640/−1350–10,720 ± 132	Callow et al. (1964), Inskeep (1962)
Enkapune Ya Muto (GtJi12)	Kenya	X	29,280 ± 540–500 ± 145	Ambrose (1998)
Ishango 11*	DRC		23,760 ± 385–19,870 ± 240	Brooks and Smith (1987)
Nasera	Tanzania	X	22,910 ± 400–7100 ± 75	Ambrose (1998), Mehlman (1977)
Lukenya Hill (GvJm-46)	Kenya		20,395 ± 1000–19,330 ± 1120	Miller (1979)
Lukenya Hill (GvJm-22)*	Kenya	X	17,670	Gramly (1976), Gramly and Rightmire (1973), Kusimba (2001)
Makubasi SE	DRC	X	18,000 ± 100	Mercader and Brooks (2001)
Laga Oda	Ethiopia	X	15,590 ± 460–325 ± 70	Brandt (1988), Clark and Price (1978), Clark and Williams (1978)
Mirsaale	Somalia		12,910 ± 180	Clark (1954)
Iwo Eleru*	Nigeria	X	11,800 ± 1700	Brothwell and Shaw (1971), Harvati et al. (2011), Stojanowski (2014)

* = Late Pleistocene human remains present

Caves and rockshelters occupy a culturally and geologically specialized niche. They represent identifiable markers on the landscape that repeatedly attract archaeological assemblage-producing humans and animals, sometimes over millennia. They also act as natural containers for accumulated deposits through rapid sedimentation, protection from surface erosion and weathering, self-sealing roof falls and other collapses, and more stable interior microclimates (Barker et al. 2005; Farrand 1985; Lundelius 2006; Straus 1979). This combination of attraction and preservation renders caves and rockshelters a major source of archaeological deposits in many parts of the world. Some insist that, because of this, caves and rockshelters represent the best opportunity for archaeology in circumstances where sites are rare (Farrand 1985, 2001a; Straus 1979).

However, these environments also possess their own complex suite of postdepositional taphonomic agents, including human occupants who would have modified and transformed the spaces in which they were living. Farrand (2001a) and Goldberg and Sherwood (2006) emphasize the importance of understanding cave sediments not just as anthropogenic sediments but as parts of larger stratigraphic frameworks that also include the deposits outside of the cave proper. Goldberg and Sherwood (2006, p. 20) also stress that the depositional and postdepositional processes in rockshelters are “different from those in caves, more akin-to open-air sites.” Rockshelters have much greater connection with the outside environment, are illuminated by daylight, and have less structural stability than caves (Farrand 2001b).

Open-air sites, on the other hand, have not been as well studied for several reasons, notably the difficulties in finding them and the disturbances to them caused by agriculture and construction (Clark 2015). In Iringa, a few Iron Age open-air smithing and smelting sites have been identified, such as Utinde Mkoga (Lyaya 2012; Lyaya and Mapunda 2014; Msemwa 2002). Ongoing research at Loiyangalani initiated by Bower (1977, 1981, 1985) demonstrates the challenges and opportunities inherent in excavating open-air sites in Tanzania.

To Pleistocene people, Mlambalasi would have been a spacious, well-protected shelter with an attractive surrounding environment. The Eastern Arc mountains, a 5400 km^2^ area extending from southern Kenya to southern Tanzania, have been relatively stable for the last 13,000 years and potentially much longer (Finch et al. 2009; Mumbi et al. 2008; Willoughby 2012). Stable carbon-isotope analysis of lake cores from the Dama Swamp, located about 70 km from Mlambalasi, indicates no change in ratios of C3 to C4 over the last 24,000 years. Even during the LGM, the immediate region would have supported a moist forest (Mumbi et al. 2008; Willoughby 2012). Cores from Deva Deva in the nearby Uluguru Mountains show similar stability in forest taxa (Finch et al. 2009). This suggests the Southern Highlands provided a refuge for human and animal populations for part or all of the most recent glacial, while nearby lowland areas of the East African Rift Valley experienced significant vegetation, hydrological, and climatic instability (Willoughby 2012). Iringa is therefore important to the search for human occupations during the terminal Pleistocene and perhaps other periods of environmental stress.

## Archaeological Fieldwork

The Iringa Region is located in the south-central portion of Tanzania on a high-level plateau approximately 1,400 m above sea level. Villages and large granitic outcrops dot the landscape, and the region is cut through by rivers and ephemeral streams. The Little Ruaha River, a tributary of the Rufiji River, traverses the region. The current vegetation is characterized as miombo woodland—a moist savannah type dominated by tall, densely spaced trees, found in areas with an annual rainfall of 75–100 mm and a long dry season (Hamilton 1982, p. 19). With dry montane forest on hills and mountains and savannah on the plains, the primary economic activities include farming, and cattle and goat herding, by Hehe and Maasai communities.

Iringa lies within the Usagaran Belt, formed 1.9–2 billion years ago, dominated by metamorphic rocks classified as quartzite and granites (Fritz et al. 2005; Government of Tanzania 2005; Hathout 1983; Sommer et al. 2003). This ancient geology gives rise to the most prominent features of the landscape—numerous kopjes, or “steep-sided piles of massive crystalline boulders” formed by the collapse of bornhardts or inselbergs (Buckle 2007, p. 141). Kopjes are of high archaeological potential as they are prominent, highly visible features of the landscape that erode into rockshelters. Our surveys of the region confirm that many of these shelters have archaeological surface scatter.

Very little archaeological fieldwork has been conducted in the Iringa Region, and that which has been conducted focused on either the Early Stone Age at Isimila and Mgongo (Cole and Kleindienst 1974; Hansen and Keller 1971; Howell 1961, 1972; Howell et al. 1962; Omi 1988) or on various other Iron Age sites (e.g., Sutton 1969). In 2000, Dr. Paul Msemwa, then Director of the National Museum in Dar es Salaam, undertook 14 days of fieldwork in the Iringa Region to “come up with sites that could help build up the general chronology” (Msemwa 2002, p. 1). Msemwa (2002) wanted to understand the cultural history of the region and placed strong emphasis on understanding the Iron Age interactions between coastal and interior populations. At Mlambalasi, Msemwa excavated a 2 x 1 m trench under the shelter overhang near the drip line, ceasing at 60 cm below surface when large pieces of roof fall obstructed further excavation. He returned to the National Museum with a representative sample of finds from the site.

**Fig. 3:**
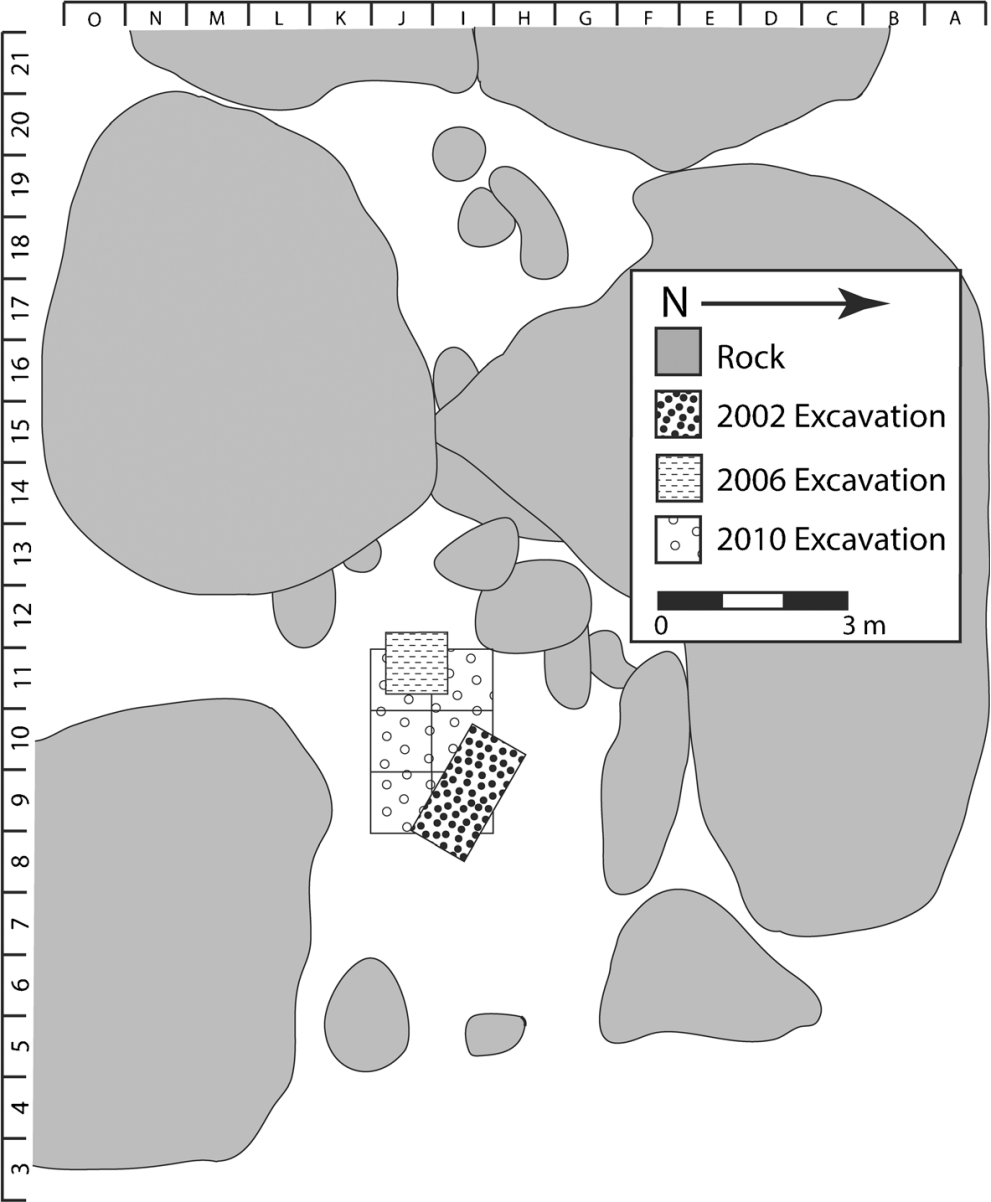
Map of Mlambalasi rockshelter showing location of the 2002, 2006, and 2010 excavations

In 2005, a local antiquities officer informed one of the authors (PW) about Mlambalasi rockshelter during an informal archaeological survey of the Iringa Region. Unaware of the previous excavation, IRAP opened two test units the following year, one under the shelter overhang and another on the slope outside the shelter (Biittner et al. 2007). By chance, IRAP’s excavations did not overlap with Msemwa’s unit (Fig. 3). The 2006 excavations by IRAP revealed a continuous, stratified sequence of occupation under the shelter overhang and disturbed deposits on the slope outside the shelter. Test pit #1 (TP1), a 2 x 1 m trench positioned centrally in room 1, was excavated to a depth of 120 cm below surface (Fig. 3). This yielded a cultural sequence with a historic/Iron Age deposit underlain by an LSA sequence (Biittner et al. 2007). Recovered in association with the LSA deposits was a partial human skeleton in a primary context burial (burial 1). Unfortunately, the test pit bisected the individual and only the lower portion of the body was recovered at that time. A small collection of faunal remains, largely representative of high-survival elements such as limbs, was also recovered (Collins and Willoughby 2010).

IRAP members returned to Mlambalasi in 2010 to recover the rest of the human burial and to determine the relative locations of the 2002 and 2006 excavation units. All matrix was screened through a 1 mm mesh, and all visually identifiable artifacts and ecofacts were collected. No artifacts were left in situ once excavated, documented, and photographed; all were collected for subsequent analyses in the lab. Six units of 1 m^2^, resulting in a 2 x 3 m trench, were excavated in 10 cm levels to a maximum depth of 110 cm below the rockshelter floor before encountering bedrock (Fig. 3). The previous excavations were relocated and mapped, and the remaining portion of burial 1 was recovered. In addition to LSA and Iron Age materials, we also recovered a German rifle casing manufactured in 1892. This find is of particular interest given Mlambalasi’s local renown as a place of colonial resistance. All material from the 2006 and 2010 excavations was exported on-loan to the University of Alberta for analysis.

## Stratigraphic Sequence and Dating

**Fig. 4:**
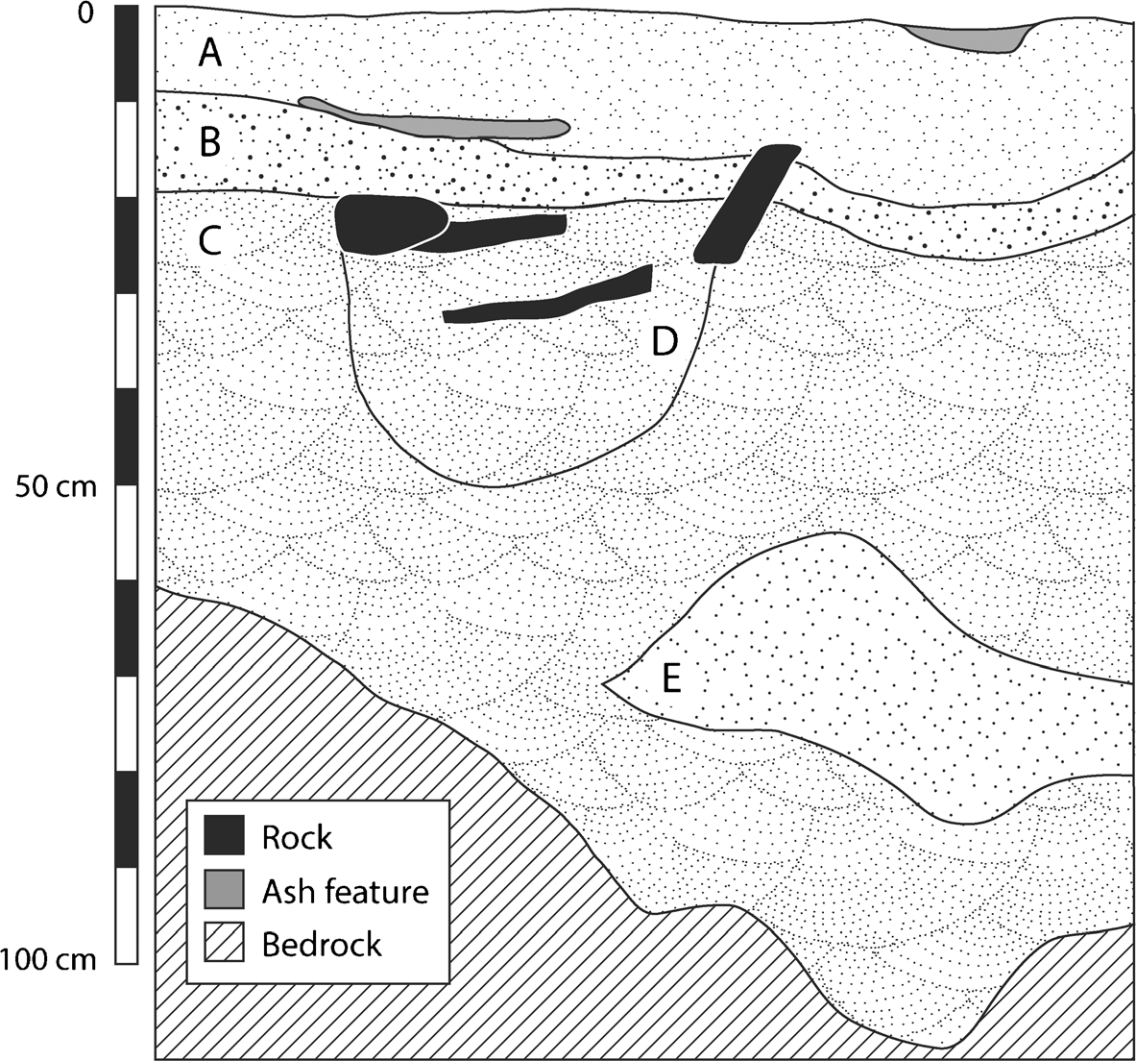
Stratigraphic profile of unit I-11, north wall

The Stone Age stratigraphy at Mlambalasi is ambiguous (Fig. 4). The sediments are made up of homogenous fine sand and silts, with varying percentages of gravel and disintegrating bedrock; however, there is a consistently large number of artifacts throughout. The only clear and highly visible changes in stratigraphy are found in the historic/Iron Age layers of the site where the smelting of iron appears to have been responsible for a discontinuous, ashy horizon (an anthropogenic sediment) captured as ash features in Fig. 4. Lithostratigraphic unit A is comprised of poorly sorted sandy silt (10 YR 3/2 very dark grayish brown). Units B and C are poorly sorted silty sand separated only by presence of some pebble- to cobble-sized inclusions in C, as they are very similar in color (10 YR 5/2 grayish brown and 10 YR 4/2 dark grayish brown, respectively). The pit feature (D) has a distinct boundary between it and lithostratigraphic unit C and contained several large rocks; however, C and D are otherwise difficult to distinguish, macroscopically, in terms of color and texture. Lithostratigraphic unit E is a lens of silty sand, lighter in color (10 YR 5/4 yellowish brown) than the other units; it also contains rootlets.

The presence of a calcium carbonate cement on most excavated materials in Stone Age deposits suggests movement of groundwater through the site at some point in the past, perhaps annually during the rainy season. Within the shelter, smaller artifacts, including faunal remains, accumulate against larger boulders that likely represent roof fall. The directionality of these accumulations approximates the overall slope of the surface at the site—suggesting movement of water from the heights of the kopje down through the relatively flat surface of the rockshelter’s interior, and finally down the slope, out of the rockshelter toward a flat plain below. This is consistent with stratigraphic disturbance of the deposits downslope.

To further complicate the stratigraphy at Mlambalasi, there is evidence of burrowing activity; the most likely culprits for bioturbation appear to be *Achatina* or giant land snails, marked by the abundance of shells in every excavation level and all stratigraphic units. The most likely candidate is *Achatina fulica*, indigenous to East Africa. It has light-brown banding on its shells, which are typically 5–10 cm long with some adult shells approaching 20 cm in length (Rowson et al. 2010; Skelley et al. 2010). *Achatina* sp. including *A. fulica* reside underground from June through November (Harrison et al. 1997). Thirteen whole shells were recovered and provenienced in the 2010 excavations. Shell fragments were not counted as they frequently break during excavation, handling, and transport; instead, the total weight per level per quadrant per unit was measured. The total weight of all recovered shells from the 2010 excavation is 5,320 g. The average weight for an *A. fulica* shell is approximately 32 g (Cooling 2005); therefore, this assemblage roughly represents the shells of at least 165 snails.

The shells vary in terms of context and condition. Those closer to the surface appear quite fresh, while others have the thick calcium carbonate coating that is common to many of the finds from deeper sediments. The context, condition, and quantity of the shell recovered suggest that snails have likely burrowed into the matrix in the recent past. It is also possible that some shells are present as the result of human activity, perhaps brought to the site as a food source (Harrison et al. 1997; Mehlman 1979). Burrowing organisms of this size may easily displace sediments and small finds. Some of the human remains also show evidence of insect burrowing (Sawchuk and Willoughby 2015), and a large termite mound was discovered just outside the rockshelter in the 2006 excavation of test pit #2. However, the high recovery of fragmentary human remains, including small elements such as the hyoid from burial 1, suggests that bioturbation agents caused minimal disturbance.

To determine the age of the deposits at Mlambalasi, a series of 14 radiocarbon samples were collected from excavations within room 1. While human and animal bones are preserved within the rockshelter, a notable exception to the pattern at most East African sites (Willoughby 2012), attempts to directly date the human bones have proven unsuccessful due to insufficient collagen. While selected dates have been previously reported (e.g., Sawchuk and Willoughby 2015; Willoughby 2012), the full suite of radiocarbon dates on *Achatina* shells, charcoal, and ostrich eggshell (OES) beads is included here (Table 2).

**Table 2:** Dates obtained on charcoal, *Achatina* sp. shell, and OES beads recovered under the shelter overhang at HwJf-02

Depth (cm)	Unit	Reference number	Material	Uncalibrated years BP	Calibrated age (95.4% prob.)
4	I-11	OxA-24622	Charcoal	151 ± 24	1470–1635 AD
12	I-11	OxA-24623	Charcoal	342 ± 24	1667–1950 AD
20	I-11	OxA-24619	Charcoal	189 ± 24	1655–1954 AD
25	TP1	TO-13416	Charcoal	460 ± 50	1405–1490 AD
40	I-11	OxA-24642	Charcoal	398 ± 24	1438–1620 AD
48	I-11	OxA-24618	Charcoal	267 ± 25	1521–1798 AD
65–70	TP1	TO-13417	*Achatina*	12,940 ± 90	13,705–13,035 BC
70	I-10 (feature B-1)	OxA-24620	Charcoal	12,765 ± 55	13,660–12,925 BC
**73**	**I-11**	**OxA-24617**	**Charcoal**	**182 ± 24**	**1660–1954 AD**
**75**	**J-11 (feature B-1)**	**OxA-24621**	**Charcoal**	**372 ± 24**	**1448–1632 AD**
75–80	TP1	OxA-27621	OES bead	14,115 ± 55	15,556–14,956 BC
80–90	I-11	OxA-27623	OES bead	14,275 ± 55	15,718–15,703 BC
90-100	I-9/J-9	OxA-27624	OES bead	16,690 ± 65	18,203–17,606 BC
**110–120**	**TP1**	**TO-13418**	***Achatina***	**11,710 ± 90**	**11,820–11,395 BC**

Note: TO dates were calibrated using INTCAL04 Terrestrial Radiocarbon Age Calibration (Reimer et al. 2004). OxA calibrations were generated using the Oxcal computer program of C. Bronk Ramsey, using the “INTCAL09” dataset (Bronk Ramsey 2009; Reimer et al. 2013). Data in bold indicate intrusive samples

Dates that should be regarded as intrusive are shaded in gray. The tunneling activity of *Achatina* sp. means that small finds may not be in primary context; a more dependable interpretation of the radiocarbon dates is to consider them as marking a minimum age. They may reflect the true age of the deposit or may significantly postdate it. With this interpretation, the oldest occupation represented under the shelter roof at Mlambalasi appears to have accumulated during the final stages of the Pleistocene. However, Grine (2016) questions whether the reversal of *Achatina* dates bracketing burial 1 suggests the remains are an intrusive Holocene burial rather than reflecting the activity of snails.

While a hearth feature was associated with Iron Age deposits, none were recovered from Stone Age levels at Mlambalasi. As such, dates obtained on isolated charcoal from Stone Age layers are also difficult to link conclusively with human activity. For example, an anomalously recent date found with the burial 1 feature (75 cm BS, 372 ± 24 BP) was recovered in a roughly triangular, loosely packed area of darker-colored sediment that contrasted with the surrounding matrix and was likely a burrow.

To address these uncertainties and establish a more precise date for the human remains, we submitted three OES beads that were recovered near burial 1. One was recovered in 2006 near the right wrist of the individual; the other two were excavated with the rest of the remains in 2010. All three dated beads were recovered between 75 and 90 cm below surface, and the dates range from 15,550 to 18,200 cal BC. These results reinforce the interpretation that the earliest occupation levels below the shelter overhang at Mlambalasi are terminal Pleistocene in age.

The 2010 field season recovered approximately 250 kg of finds during 21 days of excavation. Table 3 presents a summary of the material culture recovered for each excavation level. As stated, *Achatina* shell is provided in grams per level to account for the fragile and fragmentary nature of this material. Human remains are not included as this data is presented elsewhere (Sawchuk and Willoughby 2015). The artifacts associated with iron smithing and/or smelting include slag, finished tools and nails, and furnace and tuyere fragments, and have not yet been analyzed.

**Table 3:** Summary of the material culture recovered by level from HwJf-02 during the 2010 excavation

Level and depth below surface (cm)	Lithostratigraphic unit	Pottery	Iron tools/slag/furnace fragments	Lithics	Fauna	*Achatina* shell (g)	Glass, plastic, and OES beads; beadmaking materials (modified OES)
Surface	A	84	605	743	264	325.1	2
1 (0–10)	B	268	4,730	5,778	2,422	1,148.6	25
2 (10–20)	C	154	2,416	6,487	3,005	1,484.5	18
3 (20–30)	C	114	1,679	7,517	2,938	1,141.5	22
4 (30–40)	C	39	968	7,029	2,475	451.2	10
5 (40–50)	C	13	415	5,306	1,680	223.8	9
6 (50–60)	C	25	478	3,975	1,676	381	10
7 (60–70)	C	15	178	2,863	644	131.7	2
8 (70–80)	C	4	82	2,723	330	25.5	3
9 (80–90)	C	1	78	1,876	573	7.4	5
10 (90–100)	C	0	25	642	113	0	0
Totals		717	11,654	44,939	16,120	5,320.3	106

The highly unconsolidated sediment, taphonomic processes (such as the *Achatina* burrowing described above), and intrusions such as the pit feature (D), provide likely explanation for the presence of pottery and artifacts associated with iron smithing and/or smelting in LSA levels. Furthermore, all units except J-10 also contained backfill from previously excavated test pits (Fig. 3). There are several possible explanations for the presence of large numbers of artifacts in the refilled 2002 and 2006 test pits (Miller 2012). First, during the backfilling process, surface, wall, and baulk artifacts could have been inadvertently dropped into the unit. Second, screens were not used in the 2002 and 2006 excavations, so small-sized artifacts could have been missed. Finally, sediment and artifact movement caused by slope and ground water effects could be responsible.

## Lithic Sequence

Analysis of the 2010 lithic sequence supports the 2006 interpretation of the cultural sequence at Mlambalasi rockshelter (Biittner et al. 2007). There is a historic/Iron Age to LSA lithic sequence within the rockshelter. This includes a trend in the LSA assemblage that may represent separate Holocene and Upper Pleistocene occupations. The uppermost (Holocene) LSA is represented by the higher proportion of microlithic tools, while lithics recovered from lower levels (Upper Pleistocene) are significantly larger in overall size. We also recovered MSA lithics from the disturbed deposits on the surface of the slope outside of the main rockshelter and in test pit #2 on the slope in 2006. The MSA component is not located within the main rockshelter, but along its margins and possibly underneath a massive piece of roof fall that collapsed at some point in the past.

The uppermost LSA at Mlambalasi is primarily a microlithic quartz assemblage, containing a high proportion of backed pieces manufactured from bipolar blanks. Of the 44,939 artifacts analyzed, 93% were composed of quartz, quartzite, or rock crystal, all readily available locally; outcrops are highly visible and readily accessible throughout the Iringa Region (Biittner 2011). High quality chert, granite, and metamorphic rock types were also used but in smaller frequencies. This pattern of raw material usage did not differ substantially between excavation levels, suggesting a relatively unchanging approach to raw material acquisition and transformation over time.

**Fig. 5:**
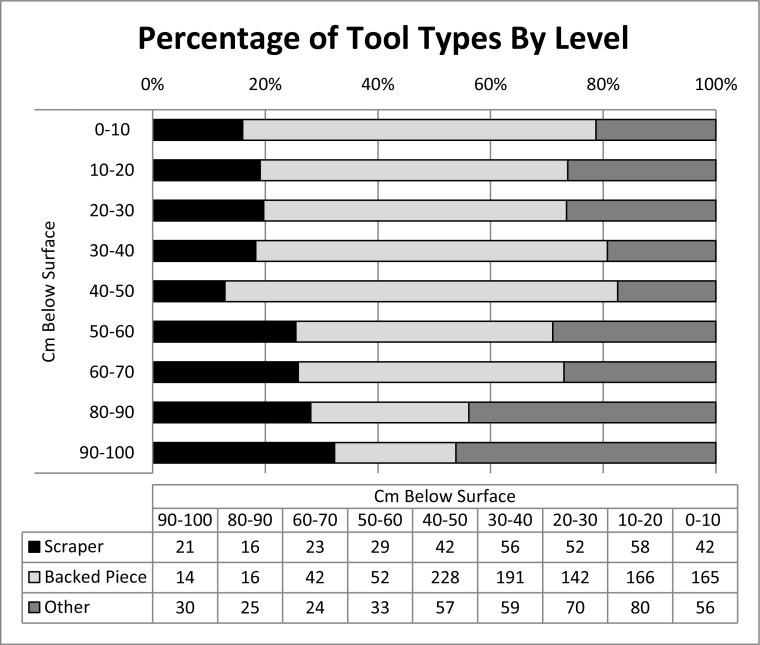
Percentage of tool types by level

Figure 5 illustrates the relative frequencies of tool types per level. Formal tools amount to approximately 4% of the total lithic assemblage and include backed pieces, scrapers, bifacially modified pieces, burins, and points. As with Biittner’s (2011) analysis of the lithic assemblage recovered in 2006, analysis of the 2010 assemblage does not demonstrate any relationship between raw material and tool type, indicating that the occupants of Mlambalasi did not routinely allocate raw materials for the manufacture of particular end-products. Easily, the largest tool category overall was backed pieces (68%), which increased in proportion through time (*r*_s_ = −0.093, *p* < 0.0001) from approximately 21% of trimmed pieces in level 9 (80–90 cm below surface) to 68% in level 1 (0–10 cm below surface). The transition was mainly at the expense of scrapers, which declined proportionally over the same period from 32 to 16%.

Other lithic artifact types present include cores, debitage, and relatively few nonflaked (i.e., raw material nodules or ground) stones. The most common flaking method was bipolar, which involves striking the objective piece from above while holding it stable on a hard anvil. The technique appears to have been deployed in response to the water-rounded shape and limited flaking quality of Iringa quartz. While bipolar technology was significantly associated with quartz reduction (*x* ^2^ = 6.879, *df* = 1, *p* = 0.017), no such relationship was observed for other raw materials or reduction strategies. Apart from bipolar cores, the presence of patterned platform and peripherally shaped cores indicates the existence of alternate reduction pathways, but these were utilized infrequently. The lack of cortex on cores (~25% remaining cortex) and a high proportion of debitage suggest that extensive core reduction/tool manufacturing was occurring at the site. Most of the debitage produced was either complete flakes/blades or angular fragments (shatter). There may have been some bias against the collection of microdebitage, as no lithic artifacts smaller than 5 mm^2^ were recovered; however, the recovery of numerous beads smaller than that size argues against a significant sampling or collection bias. Future analysis of retained sediment samples may yield microdebitage.

Lastly, a secular decline in the average size of pieces across artifact categories through time is observed. This change was significant for cores (*r*_s_ = 0.144, *p* < 0.0001), whole flakes and blades (*r*_s_ = 0.139, *p* < 0.0001), and trimmed pieces (*r*_s_ = 0.168, *p* < 0.0001). The absolute median difference in most artifacts between the top and bottom of the sequence was approximately 2–6 g. For debitage and trimmed pieces, the indicated difference in weight meant that pieces at the bottom of the sequence were frequently over twice as large as those at the top.

## Human Remains

At least three individuals were uncovered in the course of excavations at Mlambalasi rockshelter (Sawchuk and Willoughby 2015). Two commingled individuals are associated with the older LSA occupation and consist of one adult (burial 1) and one juvenile (burial 2). A second LSA adult may also be present in the fragmentary remains of this feature (burial 4). An additional set of adult skeletal material was recovered from the Iron Age horizon (burial 3) a few meters away.

**Fig. 6:**
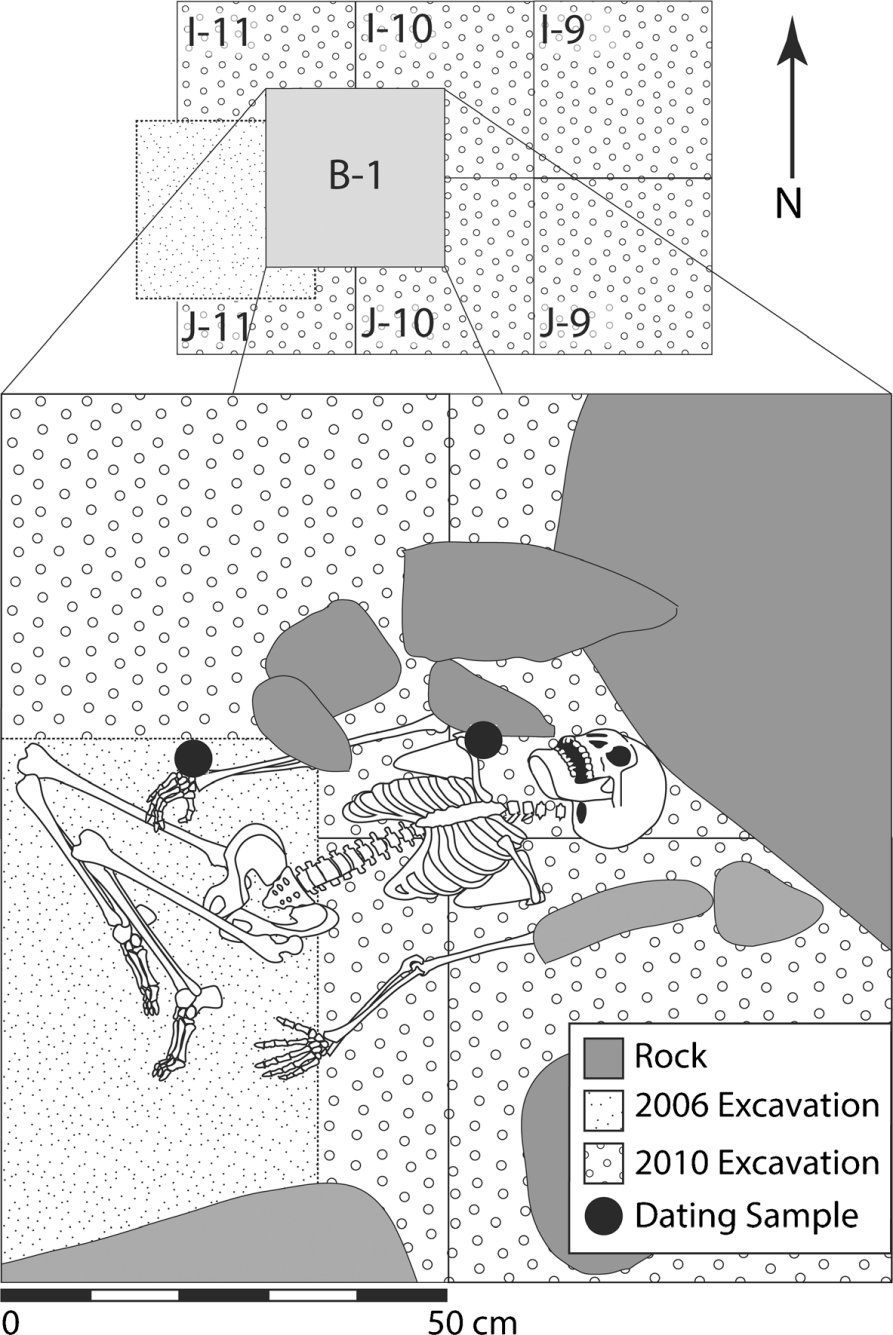
Reconstructed orientation of burial 1 at Mlambalasi

Burial 1 (B-1) is a primary in situ interment found approximately 70–90 cm below the modern ground surface, near the back of the rockshelter (Fig. 6). The individual was extended in a supine position, oriented east-west with the head at the east toward the entrance. Both arms were extended and the skull was slumped over to the right, facing the north wall of the shelter. The position of the lower body is unclear given the highly fragmentary nature of the skeleton and its recovery over the course of two field seasons, but the legs were likely flexed. Although the remains were not in a formal burial pit or cairn, the body was overlain by a series of cobbles and boulders likely originating from roof fall. The head of the individual appeared to be intentionally emplaced beneath the overhang of one of these large boulders. The body was not associated with any grave goods but was in close association with three OES beads. These beads, in addition to *Achatina* shells from above and below the remains, and charcoal found next to the right shoulder, provide a consistent date within the terminal Pleistocene.

The B-1 individual is a middle-aged adult of unknown sex. Given the poor preservation of the remains, estimated age at death is based on skeletal development and dental wear, including advanced wear on the third molars. Sexually dimorphic features on the fragmentary skull and pelvis are not diagnostic, although the gonial angle of the mandible appears masculine. Osteometric sex estimation techniques applied to the mandible (Saini et al. 2011) and metacarpals (Scheuer and Elkington 1993; Stojankowski 1999) yielded conflicting results, possibly related to the overall small size of the skeleton. Although no complete long bones are present, stature estimates based on the diameter of the femoral head (Simmons et al. 1990) and complete left first and right fifth metacarpals (Meadows and Jantz 1992) suggest a height of 155.3 to 166.3 cm with an average of 160.7 cm for a male and 150.8 to 163.2 cm with an average of 156.9 cm for a female. Body mass estimates using the maximum diameter of the femoral head were equally diminutive: 43.67 to 44.58 kg for a male and 44.58 to 50.79 kg for a female, with averages of 44.13 and 47.69 kg, respectively. Given that little is known about diversity in body size and sexual dimorphism in the terminal Pleistocene, this individual may either be a female or small male. Other notable skeletal features include a high rate of carious lesions, atypical in forager populations, and possible pathological changes on the sphenoid and petrous portion of the right temporal.

Remains of the second LSA individual (B-2) are limited to a partial juvenile manubrium, most likely from an older child. It was found within the B-1 feature, making it probable that these remains also date to the terminal Pleistocene. We also found a mostly complete, adult upper jaw with a grossly carious right lateral incisor and a large lesion representing a periapical abscess or periodontal cyst (Dias et al. 2007). Albeit taphonomically altered, the alveoli are inconsistent with the isolated dentition from B-1. The fragment therefore may represent a third LSA individual (B-4). If so, the presence of advanced dental disease in multiple specimens could indicate a broader pattern of health in this population.

The Iron Age burial (B-3) consists of a fragmentary skull, upper arms, and partial thorax of a possibly female adult. The antiquity of this skeleton is unknown, but it was found near an anthropogenic ash layer 25 cm below surface, dated to 460 ± 50 uncalibrated years BP (TO-13416) (1440 cal AD). This burial was located several meters away from the LSA individuals, near the Iron Age smelting furnace by the entrance of the rockshelter. Only a portion of the rockshelter has been excavated, so additional components of these individuals may be recovered in the course of future excavations.

## Faunal Remains

Nonhuman faunal remains are highly fragmented; 75% of the material excavated in 2006 is less than 30 mm in length and represents highly survivable skull and limb elements (Collins and Willoughby 2010). Analysis of the faunal remains from 2010 is ongoing. Identification to taxa is not possible in most cases, but the 2006 test pits show a predominance of bovid (*Bos taurus*, *Synecerus caffer*) and caprine (*Capra hircus*, *Caprini indet*.) remains from recent occupation levels. Poor preservation and small sample size preclude interpretation of subsistence behaviors in the LSA, but the frequency of cut marks, calcined and burnt bone, and heavy fragmentation are all suggestive of repeated campsite usage during the Iron Age.

## Beads

A total of 127 beads has been recovered from excavations at Mlambalasi (72 OES, 52 glass/plastic, and 3 of unidentified organic material). The vast majority was recovered in 2010, with only a single “European” bead found in 2002 (Msemwa 2002, p. 14) and 2 OES beads in 2006. Detailed analysis has thus far been directed only at the OES beads (see Miller 2012). Since the 2010 excavations overlapped the previously excavated areas, a significant portion of finds were recovered from a disturbed matrix, and only 38 OES beads can be confidently assigned to a primary context.

The most common unit of analysis for archaeological OES beads is external diameter; previous studies of the southern African hunting/herding transition found that older beads are often smaller than younger beads (e.g., Jacobson 1987a, b; Orton et al. 2005; Sadr et al. 2003; Smith et al. 1991, 1995, 2001). This diameter change also appears to be present at Mlambalasi. Thirty-one of the 38 primary context OES beads were complete enough for a diameter analysis and show a mean diameter shift of 2 mm between the upper and lower excavation layers. This sample size is very small, and some excavation levels are represented only by a single bead, which severely affects the potential range of diameters per level. Further recovery and analysis of OES beads at Mlambalasi may aid in the comparison between the East African and southern African bead diameters.

**Fig. 7:**
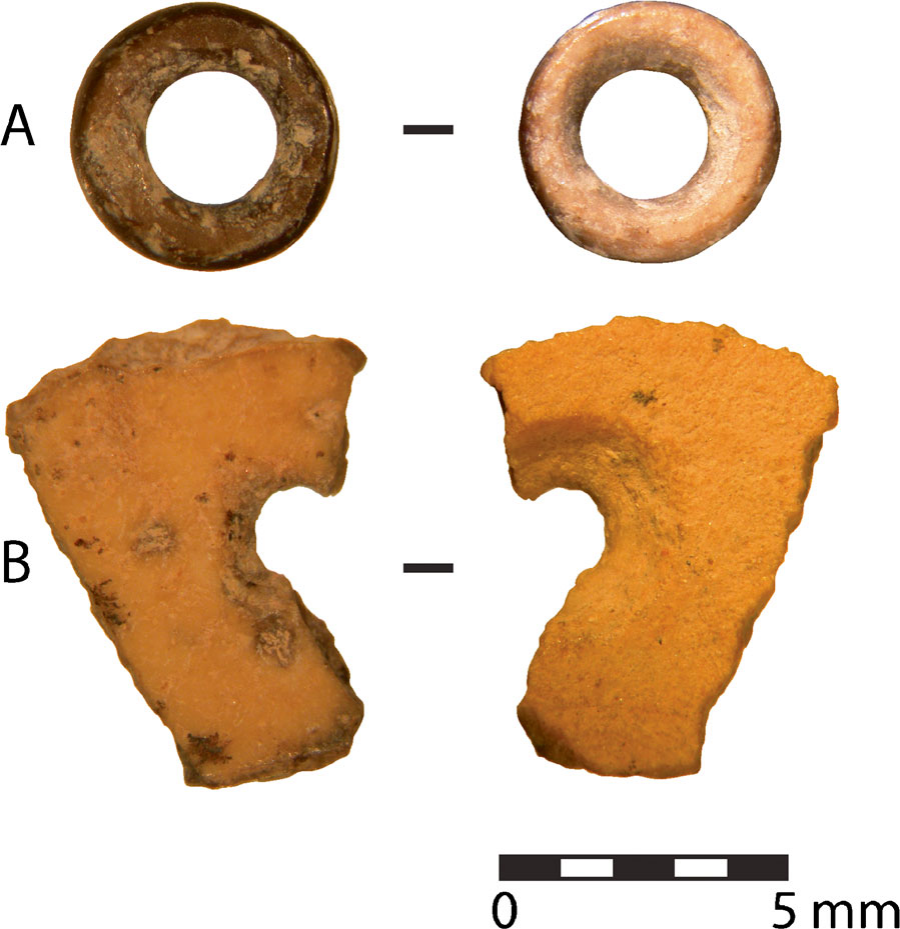
Direct dated OES beads recovered near burial 1. Cuticle surface shown on the *left*, mammillary surface shown on the *right*. Bead A (sample OxA-27624) dates to ~20,000 years ago. Bead B (sample OxA-27623) dates to ~17,000 years ago (photo: J. Miller)

The three directly radiocarbon-dated beads were recovered near the lowest excavation levels at Mlambalasi, deep within the LSA deposit. They range in age from approximately 15,500 to 18,200 cal BC. One was recovered near the right wrist of burial 1 in 2006, while the other two were found in 2010 directly below the body. Microscope photos are not available for the 2006 artifact; however, it is a completed bead with limited use wear or evidence of heating and an average external diameter of 6 mm. Bead A, shown at the top of Fig. 7, is a completed bead with a shiny patina and reddish brown color likely created by the application of heat. It is the oldest of Mlambalasi’s directly dated beads with an approximate age of 20,000 years. Bead B, shown at the bottom of Fig. 7, is a partially fabricated preform, which likely broke during manufacture. It has a completely drilled aperture and partially trimmed outer edges; these characteristics assign the bead a Kandel and Conard (2005) production value of 8 and would be classified by Orton (2008) as a pathway 1, stage 4b.

At approximately 17,000 years old, the directly dated preform is not as old as the completed bead; however, its incomplete production is a window into the manufacturing technique of Later Stone Age people. The pathway 1 technique, which involves drilling the aperture prior to outer shaping, can be significantly more time effective than other techniques and is commonly used in OES beadmaking today (Miller 2012; Wingfield 2003). This directly dated preform, along with the extremely similar preform from Magubike rockshelter which dates to 47,750 ± 750 BP (Miller and Willoughby 2014, p. 120), indicates the significant antiquity of this technique in the Iringa Region.

## Ceramic Artifacts

Ceramic artifacts were found in all six of the excavated units. The potsherds (*n* = 717) are evenly distributed across all units, with 45.8% recovered from Iron Age/historic deposits on the surface and in the first 10 cm below surface. As seen in Table 3, a sharp decrease in the number of potsherds recovered occurs after 40 cm below surface. Data collection included sherd size, condition, color, vessel part, temper size and type, and surface manipulation. Overall, the sherds are poorly preserved and small in size. Just over half the sherds (58.1%) are smaller than 2.5 × 2.5 cm and 40.7% are less than 7 × 7 cm in size. Most were body sherds, as only 6.1% clearly represented a rim, neck, shoulder, or base. Because of the small sherd size and lack of representative vessel parts, no vessel forms could be inferred.

All sherds are coarse and porous. The colors range from black to dark reddish gray in color (Munsell soil color: 5 YR 2.5/1, 5 YR 3/1, 5 YR 4/1, and 5 YR 4/2). Many show evidence of partial oxidation and/or burning; this suggests that the vessels were fired directly in a hearth versus air dried or fired in a kiln. Some of the sherds are completely black, which suggests a high inclusion of organic material as temper; however, 98% of the sherds are tempered with fine- to medium-sized sand. Since the sherds have uneven thicknesses but are relatively thick in cross section, the vessels were most likely created using a pinching/drawing technique.

**Fig. 8:**
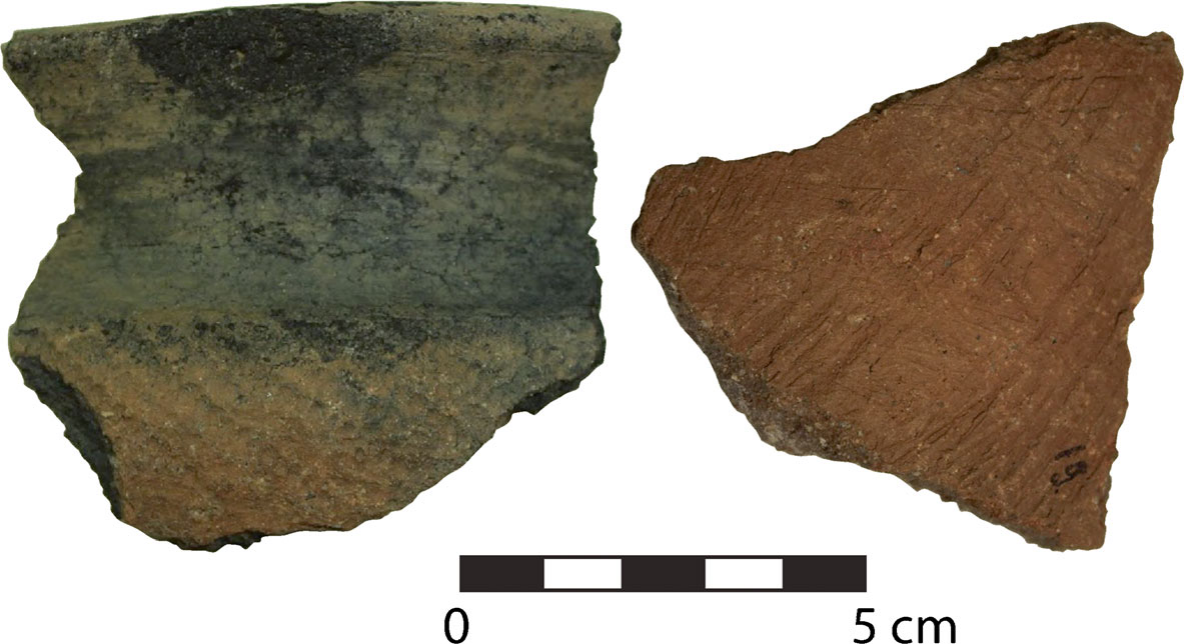
Sample of pottery from Mlambalasi demonstrating typical surface decoration (photo: J. Miller)

In terms of surface manipulation, the Mlambalasi assemblage is very similar to what has been reported elsewhere in southern Tanzania (Kwekason 2013). Overall, only 16.4% of the sherds are decorated. The frequency and types of decoration remain similar at all levels in all units; refer to Fig. 8 for examples. Most of the sherds have coarse brush strokes. After brushing, the most common decoration technique involves creation of striations through incising and/or combing. A few sherds have rouletting; patterning and shape suggests the use of a cord wrapped around a stick. Two rim sherds have perforated holes; the infrequency of this form of surface modification suggests that it was used as a repair rather than decorative technique (Sutton and Arkush 2009, p. 129).

## Rock Art

Rock art was identified on the main overhang in 2006 largely because of the modern graffiti that covers it. There are two red geometric designs and a few white dots. All are highly faded. In 2010, additional rock art was located on a granite rock surface approximately 20 m north of the main overhang. This rock art includes at least six red anthropomorphic figures and three concentrated areas of red color that are too weathered to describe more clearly.

Since 2010, additional surveys for rock art in the region revealed two more sites in close proximity (within c. 250 m) to the main rockshelter (Itambu 2013). Itambu (2013) refers to the rock art documented by IRAP in 2006 and 2010 as Mlambalasi 1 and 2. Mlambalasi 3, located about 50 m from Mkwawa’s funeral monument, is a granitic boulder with three shelters, two of which have faint red geometric paintings (Itambu 2013, p. 40). Mlambalasi 4 is an additional rockshelter located 100 m away from Mlambalasi 3 (Itambu 2013, p. 41); it has three rock facings, one of which bears a painted depiction of a long segmented body with short radiating lines, probably representing a millipede, while the other two bear faded and unidentifiable red paintings. Itambu (2013, p. 68) notes the similarity between the red geometric and anthropomorphic designs at all four Mlambalasi rock art sites and the “Hunter-Forager Figurative Fine Line” or HFFFL tradition defined by Mabulla (2005) that is predominant in central, north-central Tanzania. Similar red geometric paintings have been reported at Kisese 2 in Kondoa and in the Mara Region (Leakey 1983; Mabulla 2005).

## Discussion

The archaeological sequence at Mlambalasi is compelling because it is comparatively rare in sub-Saharan Africa and exhibits use by diverse human communities for at least 20,000 years. Furthermore, Mlambalasi is one of only 16 sites across Africa with hominin remains dated to marine isotope stage 2 (MIS 2) and one of two in eastern Africa (Grine 2016). Material culture remains within and around the rockshelter demonstrate the repeated occupation and significance of Mlambalasi rockshelter into the historic period.

Overall, the lithic assemblage is characterized by the dominance of locally available raw material and the presence of expediently produced tools with a low degree of formalization. Tools were produced, used, maintained, and discarded at the shelter. Lithic technology focused on the bipolar reduction of local quartz and quartzite to produce microliths. The use of bipolar technology is not characteristic of any period here but rather reflects raw material constraints including size, availability, abundance, and quality. Ubiquitous across and highly visible upon the modern Iringa landscape, quartz and quartzite nodules of any size readily fracture using bipolar technology, producing sharp, usable edges requiring little to no retouch to serve as functional edges (Biittner 2011). The presence of patterned platform and peripherally shaped cores indicates inherited and shared technological traits with LSA and MSA foragers (Bushozi 2011).

Although the lithic assemblage at Mlambalasi is, for the most part, characterized by continuity, there are subtle changes in typological composition and median artifact size. In particular, backed pieces increased in representation at the expense of other tool types, mainly scrapers. Kusimba (2001) discusses a common transition in eastern, southeastern, and southern LSA sites from scraper-dominated to microlith-dominated assemblages. The lithic assemblage at Mlambalasi fits this general trend and is similar to the older LSA deposits at Lukenya Hill in the predominance of scraper and flake tools and use of quartz (Kusimba 1999). The growing reliance on a single tool type may suggest that the role of backed pieces expanded to fill a larger variety of functional purposes, including armatures for hunting weapons (Bushozi 2011). Similar contemporaneous assemblages from Cameroon and the DRC indicate that this strategy was widely applicable to a number of different environments (Cornelissen 2003). Seitsonen’s (2010) analysis of Kansyore lithic technology demonstrates the flexibility and persistence of quartz-based industries over thousands of years, enduring throughout changes in mobility, subsistence, and ceramic technology. If climatic events such as the LGM were responsible for local environmental fluctuations in the area surrounding Mlambalasi, a flexible strategy that incorporated small quartz tools may have been advantageous. In addition to changes in artifact form, a systematic decrease in the size of lithic artifacts was noted between levels. This pattern may represent a refinement or intensification of the reduction process over time in response to external economic or other factors, such as technological readjustments.

The Mlambalasi lithic assemblage shares some key similarities with the nearby site of Magubike. In particular, backed pieces represent the greatest proportion of tool types and bipolar cores represent the greatest proportion of cores (Biittner 2011). Unlike Mlambalasi, Magubike has a higher number of retouched tools including scrapers, backed pieces, and points (Biittner 2011). Some variation is also seen in raw material usage. While both sites are characterized by a predominance of quartz and quartzite with cherts and metamorphic varieties present, Magubike has a greater range of variability in the number of raw material types used (Biittner 2011). The MSA assemblages at Magubike contain a greater number of higher quality raw materials, such as chert, than seen in later assemblages at Magubike or at Mlambalasi (Biittner 2011). Greater variability in MSA versus LSA assemblages further supports the idea that the flexible, small quartz-based technological strategies of LSA peoples were an adaptive advantage.

There is a suggested link between OES bead assemblages and the manner of site occupation (but also see Wilmsen 2015); when employing interpretive models by Jacobson (1987a) and Wadley (1989), the Mlambalasi beads are consistent with a short-term dispersal camp. Although Mlambalasi contains a few early bead-manufacturing stages (*n* = 14), it is skewed toward completed beads. This suggests that some casual manufacture was happening on site, and the notable absence of nonartifactual OES confirms that large-scale production was unlikely to be taking place. It appears that people at Mlambalasi were afforded the free time for bead production but did not invest heavily in it as a means of decoration, gifting, or communication. The OES evidence indicates a Stone Age occupation of Mlambalasi by a small group with limited need for highly symbolic or ritualized behavior (Wadley 1989, p. 43).

Mortuary behavior in the rockshelter appears opportunistic. Despite the presence of at least three individuals, Mlambalasi does not fit the paradigm of a formal cemetery, defined by Hall (2000, p. 140) as “a significant number of contiguous burials with a sense of boundedness related to some landform, where burial density falls off rapidly at the edge.” There are no indications that any of the past occupations served a primary mortuary purpose; the remains are neither contiguous nor significant in density. Although rockshelters provide a natural sense of boundedness, this is also why they attract humans who use them for varied and fluid purposes (Barker et al. 2005; Pannell and O’Connor 2005; Straus 1979). Rockshelter burials are well known in many regions of Africa, and incidences of sporadic mortuary use by hunter-gatherers occur at places such as Gogoshiis Qabe, Somalia (Brandt 1988); Kinto/Strauss (Bräuer 1981); Kisese II (Ambrose 1998); and Mumba-Hohle in Tanzania (Bräuer 1980; Mehlman 1989), and numerous sites in southern Africa (Morris 1992). This is reasonable, given that rockshelters are attractive habitation spaces and many cultures inter their dead beneath residential floors (Adams and King 2011). At least two interment periods during the LSA and Iron Age are represented at Mlambalasi, consistent with the multiple cycles of use and abandonment in line indicated by other forms of material culture.

Rockshelters and caves represent an important avenue for continued research on the African Stone Age. Their attraction for human groups and potential for preservation may result in the only records we have of certain periods in human evolution. Mlambalasi has remained a fixed resource for the needs of various peoples over the past 20,000 years—as a place to make beads, process food, and even bury the dead. More recently, during the Iron Age, there was a shift to more dedicated use: iron smelting in an effective natural wind shelter. Yet the fluidity of this space continues; Chief Mkwawa used it as an outpost in a colonial conflict, and today, contemporary Maasai children use it as a corral for goats. Rockshelters such as Mlambalasi continue to serve many functions for a variety of passing travelers, now as in the past.

## Conclusions

The Mlambalasi rockshelter site is exceptional in its long archaeological sequence, rich material culture, and unusual preservation of organic material including faunal and human remains. Excavations in 2002, 2006, and 2010 revealed stratified LSA, Iron Age, and historic deposits and established a chronology for the site. Analysis of various occupational debris suggests the site was largely used as a campsite for nomadic LSA hunter-gatherers and Iron Age agropastoralists, serving a variety of purposes, including mortuary, through time.

Research on the Mlambalasi site, and the Iringa Region, is ongoing. Limitations of the study include the complex stratigraphic nature of the deposits and the highly fragmentary nature of many artifacts, particularly human and animal bones, as a result of taphonomic factors including periodic roof fall and trampling. Additionally, iron slag and other smelting debris at the site are still in need of focused study. Nevertheless, the site makes an important contribution to our knowledge of this region and time period.

The potential for Iringa archaeological sites to further bigger questions on human survival through the last phases of the Pleistocene and into the Holocene makes this region promising for further study. The sheer number of unexplored rockshelters offers numerous opportunities to discover well-preserved archaeological deposits from the last several thousand years to potentially much earlier. Additional survey, sampling, and excavation planned for future field seasons will allow us to address the current limitations in our understanding of the archaeological record of Iringa Region. We are particularly optimistic in light of the palaeoclimatic evidence indicating that this region may have remained inhabitable, at least throughout the LSA. Given the dearth of archaeological sites dated to the terminal Pleistocene, particularly those with preserved human remains, it is clear that Mlambalasi should be recognized for its archaeological significance as well as its role in Tanzania’s more recent history.
